# Vacuole Membrane Protein 1 (VMP1) Restricts NLRP3 Inflammasome Activation by Modulating SERCA Activity and Autophagy

**DOI:** 10.21203/rs.3.rs-2508369/v1

**Published:** 2023-01-27

**Authors:** Stephanie R. Zack, Roman Nikolaienko, Ben Cook, Ronald Melki, Aleksey V. Zima, Edward M. Campbell

**Affiliations:** Loyola University Chicago; Loyola University Chicago; Loyola University Chicago; Institut Francois Jacob (MIRCen), CEA, CNRS, Fontenay-aux-Roses; Loyola University Chicago; Loyola University Chicago

**Keywords:** VMP1, inflammasome, NLRP3, dysfunctional mitochondria, SERCA, autophagy, α-synuclein

## Abstract

Altered expression of vacuole membrane protein 1 (VMP1) has recently been observed in the context of multiple sclerosis and Parkinson’s disease (PD). However, how changes in VMP1 expression may impact pathogenesis has not been explored. Here, we report that genetic deletion of VMP1 from a monocytic cell line resulted in increased NLRP3 inflammasome activation and release of proinflammatory molecules. Examination of the VMP1 dependent changes in these cells revealed that VMP1 deficiency led to decreased SERCA activity and increased intracellular [Ca^2+^]. We also observed calcium overload in mitochondria in VMP1 depleted cells, which was associated with mitochondrial dysfunction and release of mitochondrial DNA into the cytoplasm and the extracellular environment. Autophagic defects were also observed in VMP1 depleted macrophages. Collectively, these studies reveal VMP1 as a negative regulator of inflammatory responses, and we postulate that decreased expression of VMP1 can aggravate the inflammatory sequelae associated with neurodegenerative diseases like PD.

## Introduction

Vacuole membrane protein 1 (VMP1) was first characterized in models of acute pancreatitis [1]. This initial study observed that VMP1 regulates autophagosome formation and autophagic flux, suggesting that it promotes cellular homeostasis [1]. VMP1 also facilitates lipid droplet formation, and most recently has been found to be a crucial host factor for SARS-CoV-2 and pan-coronavirus infection [1–4]. Interestingly, VMP1 modulates contacts between the endoplasmic reticulum (ER) and mitochondria, lipid droplets, and endosomes through its interaction with the sarcoplasmic/endoplasmic reticulum calcium ATPase (SERCA), such that the interaction between VMP1 and SERCA modulates cytosolic Ca^2+^ levels close to these membrane contacts [5, 6]. However, despite being first identified within the context of an inflammatory disease, the role of VMP1 in regulating cellular inflammatory responses has not previously been investigated.

Inflammatory responses are necessary and beneficial for controlling infections but need to be tightly regulated to prevent unnecessary damage to the host. Dysregulated or excessive inflammatory responses can contribute to several diseases including neurodegenerative diseases like Alzheimer’s disease (AD) and Parkinson’s disease (PD) and autoimmune disorders such as multiple sclerosis (MS) [7–9]. Inflammatory responses are often mediated by the inflammasome, a multiprotein complex that is assembled and activated by numerous sterile and pathogen derived stimuli [10]. Notably, inflammasome activation can be regulated by changes in cytoplasmic ion concentrations and alterations in autophagy [11, 12].

Inflammasome activation consists of a ligand-sensing protein such as nucleotide-binding domain, leucine-rich repeats containing family, pyrin domain-containing-3 (NLRP3), an adaptor protein such as apoptosis-associated speck-like protein containing a CARD (ASC), and finally an effector caspase, most commonly caspase-1 [13, 14]. Assembly of the inflammasome results in the activation of caspase-1, a protease which then cleaves proinflammatory cytokines such as proIL-1β and gasdermin d (GSDMD) which induces the formation of pores in the plasma membrane through which IL-1b and other inflammatory effectors are released [15–17]. Canonical inflammasome activating signals include reactive oxygen species (ROS) overproduction, K^+^ efflux, mitochondrial dysfunction, ER stress, and lysosomal rupture [18–21]. Although Ca^2+^ flux is not thought to be directly sensed and induce inflammasome activation, it is known that changes in cytoplasmic [Ca^2+^] can induce K^+^ efflux and mitochondrial dysfunction, which can both directly activate the inflammasome. Given the putative connection between VMP1 function and inflammasome activation, and the recent connection between VMP1 and inflammatory diseases, we hypothesized that dysregulation in VMP1 KO cells may exacerbate cellular inflammatory responses, particularly related to inflammasome activation [22].

Here we show that VMP1 restricts NLRP3 inflammasome activation and the release of proinflammatory cytokines in THP-1 cells, a commonly used model of macrophages and microglia. We show that VMP1 knockout (KO) leads to increased release of IL-1β and other inflammatory mediators following lipopolysaccharide (LPS) and alpha-synuclein (α-syn) fibril treatment. In VMP1 KO cells, we observe elevated levels of cytosolic Ca^2+^ in response to the activation of Ca^2+^ dependent signaling pathways and a corresponding reduction of Ca^2+^ stores in the ER. We also observed that mitochondria in VMP1 KO cells exhibit evidence of Ca^2+^ overload and mitochondrial dysfunction, accompanied bythe exposure of mitochondrial DNA (mtDNA) in the cytoplasm and the extracellular environment. We also observe disruption of autophagic flux in VMP1 KO cells, which may limit the ability of cells to degrade damaged mitochondria and thereby exacerbate inflammatory responses. Collectively, these results reveal a previously unreported role for VMP1 in regulating cellular inflammatory responses and provides a mechanism by which changes in VMP1 expression during disease may influence disease pathogenesis.

## Results

### VMP1 depletion exacerbates the release of proinflammatory molecules.

**Figure 1:**
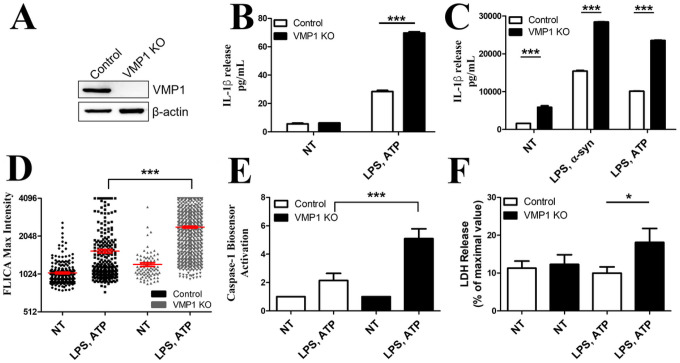
VMP1 attenuates the release of IL-1β and NLRP3 inflammasome activation under inflammatory conditions. THP-1 cells were depleted of VMP1 by using CRISPR-Cas9 genome editing. A) Protein expression in the depleted cells was confirmed by western blotting. B) Differentiated control or VMP1 KO THP-1s were treated with 100 ng/mL LPS for 4 h and 5 mM ATP for 30 min. IL-1β released into the supernatant was measured by ELISA. C) Differentiated control or VMP1 KO THP-1s were treated with 100 ng/mL LPS for 4 h then 1 μM α-syn fibrils for 24 h or 100 ng/mL LPS for 4 h and 5 mM ATP (30 min). Supernatant was collected at the same time for all treatments, and IL-1β released into the supernatant was measured by ELISA. D) Caspase-1 activation was assessed by confocal microscopy using a FLICA assay. Data are represented as the intensity max per cell from 10 images per treatment. E) Control or VMP1 KO THP-1s transduced with a caspase-1 biosensor were activated with LPS and ATP and luminescence was measured at 7.5 h following treatment. F) Control or VMP1 KO THP-1s were treated with LPS and ATP and cell death was measured by LDH assay. Data are shown as mean ± SEM and are either representative of or the average of at least three independent experiments. Statistical differences were calculated with two-way ANOVA followed by Bonferroni post-test. For all statistical tests, *,**, ***, *p* < 0.05, 0.01, and 0.001, respectively.

To determine whether VMP1 affects the secretion of proinflammatory cytokines and other molecules, we utilized CRISPR-Cas9 to knockout VMP1 in THP-1 s, a human monocytic cell line that can be terminally differentiated into macrophage-like cells [4, 23]. The knockout was verified by western blot (Fig. 1A). To activate inflammatory pathways, differentiated THP-1s were first primed with LPS then ATP to activate the NLRP3 inflammasome. Treatment of VMP1 KO THP-1s with LPS and ATP resulted in increased IL-1β secretion compared to treated control cells, suggesting an increase in inflammasome activation in VMP1 KO cells (Fig. 1B). VMP1 mRNA expression levels did not change following LPS/ATP treatment (Fig. S1A), although pro-IL-1b mRNA levels were increased in VMP1 KO cells following LPS and ATP treatment (Fig S1B). We and others have also observed that a-syn fibrils can induce NLRP3 inflammasome activation via their ability to induce vesicular damage following endocytosis [24–26]. We therefore wanted to determine whether primed VMP1 KO macrophages treated with α-syn fibrils release more IL-1β than control cells. For these treatment conditions, supernatant was collected after 24 hrs, and IL-1β release was measured. After 24 hours in culture, there was elevated basal IL-1β release from VMP1 KO cells compared to control that was not apparent after a 30 min culture, suggesting that VMP1 KO cells exhibited basal inflammasome activation without the addition of inflammatory stimuli (Fig. 1C). Primed VMP1 KO cells treated with α-syn release more IL-1β than control cells (Fig. 1C). We also measured the release of galectin-3 (Gal3), another inflammatory protein that depends on GSDMD pores for its secretion [27]. VMP1 KO cells also had increased Gal3 release from cells treated with LPS and ATP (Fig. S1C) while we observed no significant change in the expression of gal-3 mRNA in VMP1 KO cells (Fig. S1D). Overall, these data suggest that both at a basal level as well as following activation by inflammatory stimuli, VMP1 KO cells release more proinflammatory molecules.

### VMP1 KO cells have increased NLRP3 inflammasome and caspase-1 activation.

To determine whether VMP1 KO cells have increased inflammasome activation, we employed a fluorochrome-labeled inhibitors of caspases (FLICA) assay to measure active caspase-1. Treatment of VMP1 KO cells with LPS and ATP resulted in an increase in FLICA signal compared to control cells in both cell types (Fig. 1D). However, higher levels of FLICA staining were observed in VMP1 KO cells (Fig. 1D), revealing increased caspase-1 activation in these cells. Similar results were observed when caspase-1 activation was measured using a luciferase-based caspase-1 biosensor [28] (Fig. 1E). Collectively, these data suggest that VMP1 depletion increases caspase-1 activation following treatment with LPS and ATP We also observed an increase in LDH release from VMP1 KO cells, consistent with increased pyroptotic cell death, following LPS and ATP treatment, while we observed no increase in LDH release from control cells following addition of LPS and ATP (Fig. 1F). Collectively, these data suggested that in VMP1 KO sensitizes cells to inflammasome activating stimuli.

### VMP1 depletion leads to changes in genes associated with signaling and degradative pathways.

**Figure 2:**
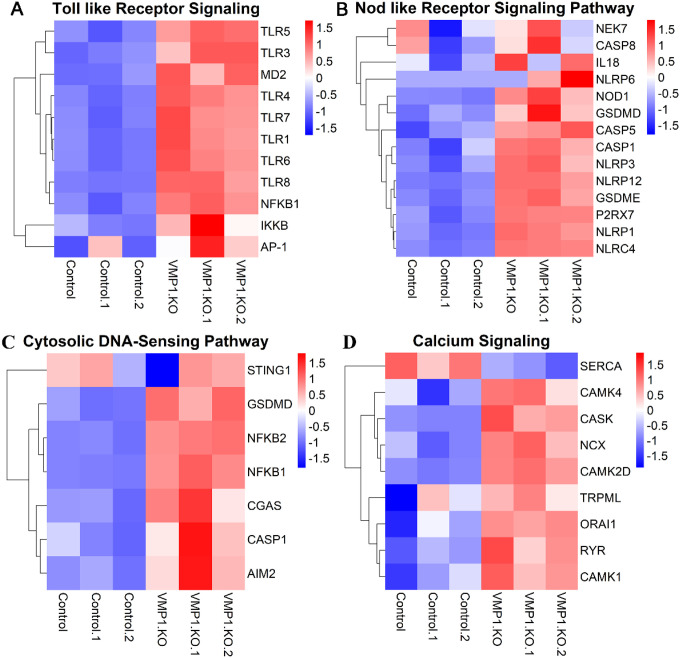
VMP1 KO leads to upregulation of innate immune pathways and calcium signaling. RNA from differentiated control or VMP1 KO THP-1 cells was isolated and relative gene expression and pathway analysis was measured using RNA-Seq. A) Heatmap showing changes in gene expression between untreated control and VMP1 KO cells for genes associated with Toll-like receptor signaling. B) Heatmap showing changes in gene expression between untreated control and VMP1 KO cells for genes associated with Nod-like receptor signaling. C) Heatmap showing changes in gene expression between LPS and ATP treated control and LPS and ATP treated VMP1 KO cells for genes associated with Cytosolic DNA-sensing pathway. D) Heatmap showing changes in gene expression between untreated control and VMP1 KO cells for genes associated with Calcium signaling. The data were analyzed following a workflow that displayed upregulated and downregulated genes in a KEGG pathway. Heatmaps were generated from the results of this analysis.

To understand the changes in VMP1 KO cells that may prime them to respond to inflammasome activating stimuli, we used RNA-Seq to define changes in gene expression that occur in VMP1 KO cells. VMP1 KO cells exhibited altered gene expression of genes associated with Toll-like receptor (TLR) signaling and Nod-like receptor (NLR) signaling pathway, observing upregulation of both pathways (Fig. 2A, 2B), consistent with our observation that VMP1 KO increased inflammasome activation and IL-1b release in these cells. Most TLRs were upregulated in untreated VMP1 KO cells compared to control cells suggesting that VMP1 KO cells were broadly primed to recognize most pathogen associated molecular patterns (PAMPs) (Fig. 2A). Furthermore, the P2X7R was upregulated in VMP1 KO cells. ATP binds to and activates the P2X7R to promote K^+^ efflux and downstream NLRP3 inflammasome activation, suggesting that VMP1 KO cells are also primed to respond to damage associated molecular patterns (DAMPs) (Fig. 2B). Several sensors that ultimately form the inflammasome complex as well as targets of caspase-1 cleavage were also upregulated in VMP1 KO cells (Fig. 2B). Upregulation of several TLR and NLR pathway genes highlight that VMP1 KO cells are primed to respond to various proinflammatory stimuli. We also observed alterations in the related Cytosolic DNA-sensing pathway when comparing LPS and ATP treated VMP1 KO cells to treated control cells (Fig. 2C). Interestingly, upregulation of gene expression in this pathway suggest that VMP1 KO cells may be more sensitive to exposed cytoplasmic mtDNA which may be increased in VMP1 KO cells, consistent with previous reports demonstrating that VMP1 mediates the selective degradation of damaged mitochondria by mitophagy [29]. We also observed changes in gene expression in genes associated with calcium signaling, including a downregulation of SERCA and upregulation of other genes associated with calcium signaling in VMP1 KO cells (Fig. 2D), consistent with the previously reported association of VMP1 and SERCA [30]. VMP1 KO cells also exhibited changes in genes associated with the autophagic/lysosomal pathway (ALP) (Fig S2A, S2B), also consistent with prior reports of VMP1 modulating activity of this pathway. Collectively, these changes to inflammatory signaling pathways and other pathways, such as calcium and the ALP suggest plausible mechanisms by which the cellular response to inflammatory stimuli may be enhanced.

### VMP1 KO cells have depleted ER Ca ^2+^ stores but elevated intracellular [Ca^2+^] with ATP activation.

**Figure 3:**
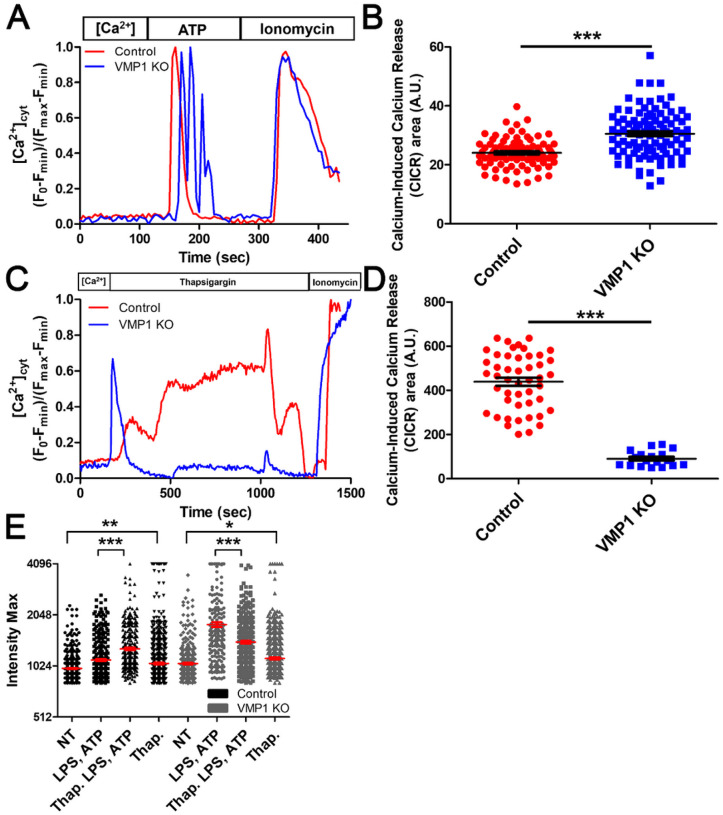
VMP1 KO cells have depleted ER Ca^2+^ stores, but increased intracellular [Ca^2+^] with ATP treatment. Either control or VMP1 KO THP-1s were perfused with Tyrode’s solution containing 3 mM Ca^2+^ for 1 min then were either perfused with A-B) ATP or C-D) thapsigargin prior to adding F_max_ consisting of Tyrode’s solution with 3 mM Ca^2+^ and ionomycin. A,C) Representative traces of the normalized fluorescence intensity of individual cells. B,D) Average calcium-induced calcium release (CICR) area under the curve of individual cells for either B) 150 sec or D) 900 sec. E) Caspase-1 activation assessed using confocal microscopy and a FLICA assay. Data are represented as the intensity max of individual cells for a given treatment. Statistical differences were calculated with two-tailed unpaired t-tests or one-way ANOVA followed by Bonferroni post-test. For all statistical tests, *, **, ***, *p* < 0.05, 0.01, and 0.001, respectively.

Changes to cytoplasmic [Ca^2+^] are known to influence inflammasome activation [31]. To measure signaling induced changes in cytoplasmic [Ca^2+^] ([Ca^2+^]_cyt_) in VMP1 KO cells, a high-affinity Ca^2+^ indicator, Fluo-4 AM, was used to monitor changes in [Ca^2+^]_cyt_ in living cells. To assess whether there were changes in [Ca^2+^]_cyt_ when cells were activated with ATP differentiated VMP1 KO or control THP-1 cells were perfused with a solution of ATP in Tyrode’s solution containing Ca^2+^. The data showed that there was an increase in [Ca^2+^]_cyt_ in VMP1 KO cells compared to control (Fig. 3A–B). Previous studies have demonstrated that VMP1 can regulate the activity of SERCA, a Ca^2+^-ATPase that transports calcium from the cytosol into the ER [5]. To measure the relative amount of Ca^2+^ stored in the ER of VMP1 KO versus control cells, differentiated THP-1s were perfused with a solution containing thapsigargin, a noncompetitive inhibitor of SERCA, in Tyrode’s solution without Ca^2+^. These data showed that there was a small amount of Ca^2+^ stored in the ER of VMP1 KO cells that was rapidly released upon the addition of thapsigargin compared to control cells that comparatively have a sustained release of Ca^2+^ (Fig. 3C–D). To test whether the VMP1 regulation of SERCA contributed to inflammasome activation, cells were treated with inflammatory stimuli in the presence of thapsigargin or thapsigargin alone. The data showed that there was increased caspase-1 activation in control THP-1s when cells were treated with LPS and ATP in the presence of thapsigargin, but not in VMP1 KO cells suggesting that inhibition of SERCA was contributing to the exacerbated inflammatory phenotype observed in VMP1 KO cells (Fig. 3E). Interestingly, when VMP1 KO cells were treated with LPS and ATP in the presence of thapsigargin, there was a decrease in caspase-1 activation, such that both wt and VMP1 KO cells exhibited similar levels of caspase-1 activation in the presence of thapsigargin following LPS and ATP stimulation (Fig. 3E). Taken together, these data reveal that ATP stimulation led to increased [Ca^2+^]_cyt_ in VMP1 depleted cells compared to treated control cells. Differences between VMP1 KO and control cells following treatment with thapsigargin suggested that the increased inflammasome activation in VMP1 cells was due, at least in part, to inhibition of the SERCA pump.

### VMP1 KO cells have an increased rate of Ca ^2+^ influx into mitochondria upon ATP stimulation.

**Figure 4:**
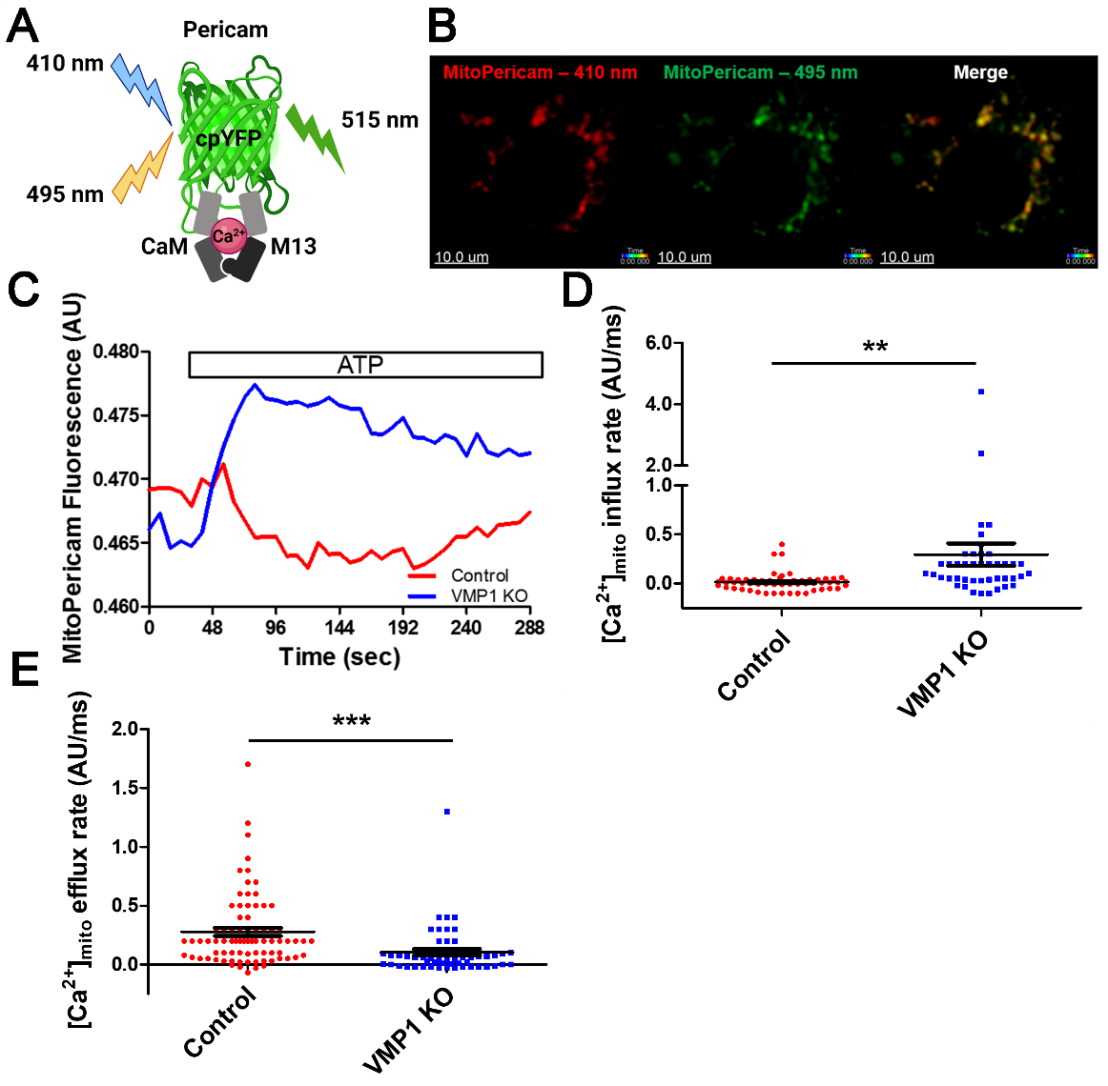
VMP1 KO cells have an increased rate of Ca^2+^ influx into mitochondria following ATP stimulation. Differentiated control or VMP1 KO THP-1s were transduced with a pMSCVpuro-Mito-Pericam construct. Cells were excited at 410 nm and 495 nm first to collect baseline recordings then cells were activated with ATP A) Schematic illustrating how the pMSCVpuro-Mito-Pericam construct works. The ratiometric pericam is a fluorescent [Ca^2+^] probe that contains a modified permutated EYFP flanked by calmodulin and an M13 peptide that contains the CaM binding site. At low [Ca^2+^], the construct is excited at 410 nm, but an increase in [Ca^2+^] facilitates the binding of CaM to the M13 peptide resulting in structural changes that cause a shift of the excitation maximum from ~410 nm to ~495 nm. B) Representative images of cells expressing the Mito-Pericam construct. C) Representative traces of the ratio of fluorescence intensity at 495 nm/410 nm of individual cells. D) Average [Ca^2+^]_mito_ influx rate of individual cells over 15 sec. E) Average [Ca^2+^]_mito_ efflux rate of individual cells over 50 sec. Data are either representative of three independent experiments (C) or the average of individual cells from at least three independent experiments. Statistical differences were calculated with two-tailed unpaired t-tests. For all statistical tests, *, **, ***, p< 0.05, 0.01, and 0.001, respectively.

It is known that mitochondria take up Ca^2+^ and buffer changes in [Ca^2+^]_cyt_ [32]. To assess how elevated [Ca^2+^]cy, in VMP1 KO cells affected mitochondrial uptake of Ca^2+^, control or VMP1 KO cells were transduced with a ratiometric pericam construct that measures changes in mitochondrial [Ca^2+^] ([Ca^2+^]_mito_) [33]. At low [Ca^2+^]_mito_, the construct is excited at ~ 410 nm, but when [Ca^2+^]_mito_ increases the pericam protein undergoes a conformational change that changes its excitation wavelength to ~495 nm (Fig. 4A). Representative images of a cell that was excited at ~ 410 nm and ~495 nm demonstrated the expression of the Mito-Pericam construct (Fig. 4B). Representative traces of the ratio of fluorescence intensity at ~ 495 nm/~410 nm for VMP1 KO or control cells treated with ATP indicated that there was a larger influx of Ca^2+^ into the mitochondria of VMP1 KO cells and that these levels were sustained over time compared to control cells (Fig. 4C). The [Ca^2+^]_mito_ influx rate was increased in VMP1 KO cells, but the [Ca^2+^]_mito_ efflux rate was decreased, suggesting that the mitochondria in VMP1 KO cells have elevated levels of Ca^2+^ for an extended period of time when cells were treated with ATP (Fig. 4D–E). Elevated levels of mitochondrial Ca^2+^ can cause Ca^2+^ overload and lead to loss of mitochondrial membrane potential [34].

### LPS and ATP treatment leads to increased mitochondrial depolarization in VMP1 KO cells.

**Figure 5:**
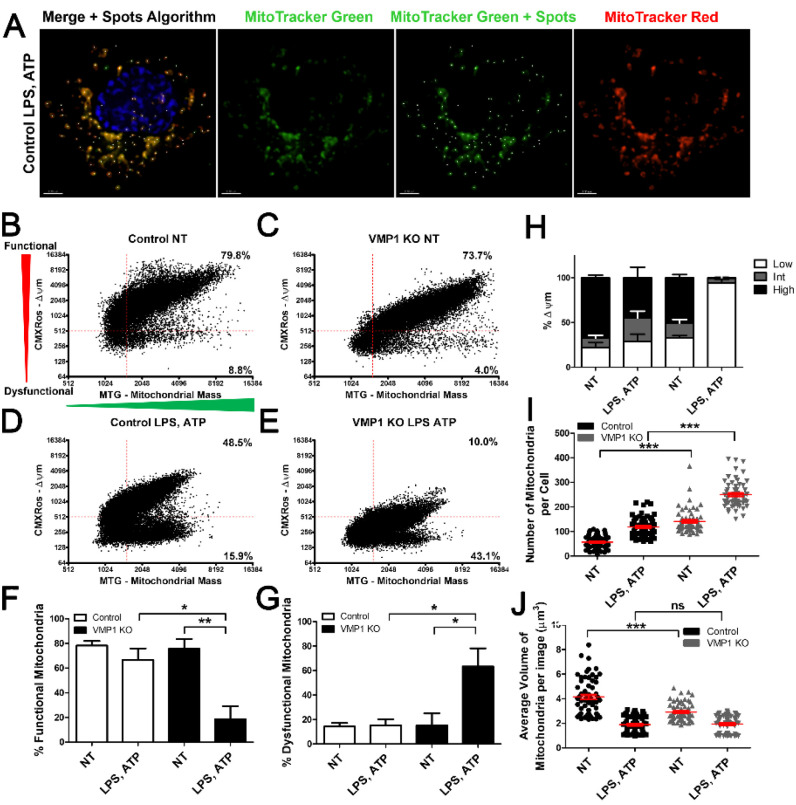
VMP1 KO cells have more mitochondria and almost all lose their membrane potential with LPS and ATP treatment. Control or VMP1 KO cells were left untreated or treated with LPS and ATP Live cells were stained with MitoTracker Red CMXRos (MTRed) with its accumulation in the cell dependent on mitochondrial membrane potential and with MitoTracker Green FM (MTGreen) to assess mitochondrial mass. Imaris was then used to build a spots algorithm around the MTGreen channel. A) Representative image of treated control THP-1s that were incubated with MTRed and MTGreen. B-E) Plots of the intensity max of MTRed indicative of the mitochondrial membrane potential vs. MTGreen indicative of the mitochondrial mass for individual mitochondria. Percentages in the upper right quadrant correspond to functional mitochondria and percentages in the lower right quadrant correspond to dysfunctional mitochondria. F) Percentages for MTRed High/MTGreen High corresponding to functional mitochondria represented as the mean ± SEM for three independent experiments. G) Percentages for MTRed Low/MTGreen High corresponding to dysfunctional mitochondria represented as the mean ± SEM for three independent experiments. I) Quantification of the number of spot surfaces indicating the number of mitochondria identified per cell, error bars represent SEM of at least 60 cells. H) Quantification of the average volume of individual mitochondria per image. Statistical differences were calculated with one-way ANOVA followed by Bonferroni post-test. For all statistical tests, *, **, ***, p < 0.05, 0.01, and 0.001, respectively.

To characterize mitochondrial function in VMP1 KO cells, we developed an imaging approach where live differentiated THP-1s were incubated with MitoTracker Red CMXRos (MTRed) and MitoTracker Green FM (MTGreen). Others have used flow cytometry based methods to perform an assessment of mitochondrial function in cells [35]. To develop a conceptually similar assay that measures mitochondrial function at the level of individual mitochondria, we used fluorescence microscopy and post-acquisition analysis to build surface masks around the MTGreen channel to allow us to measure the membrane potential of populations of individual mitochondria in these cells, as well as quantify the number of mitochondria per cell and the average volume of mitochondria (Fig. 5A, S3). To assess the relative amounts of functional or dysfunctional mitochondria, the intensity max of the MTRed was plotted on the y-axis and the intensity max of the MTGreen was plotted on the x-axis. The vast majority of mitochondria in untreated control cells were functional (high MTRed signal), and with treatment, the number of MTRed positive mitochondria decreased and an increase in MTRed negative mitochondria was observed (Fig. 5B, 5D). The relative intensity of MTRed + mitochondria also decreased following LPS and ATP treatment (Fig. 5B, 5D). Untreated VMP1 KO cells had similar mitochondria populations to control cells (Fig. 5C). However, LPS and ATP treatment induced a more pronounced decrease in the number of functional mitochondria and those mitochondria that remained MTRed + by our gating criteria had an even lower fluorescence intensity than mitochondria from control cells following LPS and ATP treatment (Fig. 5E). Data from three experiments revealed a significant decrease in functional mitochondria (Fig. 5F) and a corresponding increase in the number of dysfunctional mitochondria (Fig. 5G) in VMP1 KO cells following LPS and ATP treatment. A reduction in membrane potential was also observed in VMP1 KO cells when mitochondria were analyzed independently of MTGreen signal (Fig. 5H). Analysis of the total number of mitochondria per cell revealed that LPS and ATP treatment increased the number of mitochondria per cell, as did VMP1 KO, such that treatment of control THP-1s with LPS and ATP resulted in an increase in the number of mitochondria that was comparable to VMP1 KO cells. The average number of mitochondria per cell was highest for VMP1 KO cells treated with LPS and ATP (Fig. 5I). We observed a corresponding change in mitochondrial volume where control THP-1s have the largest and most variability in mitochondrial volume, with reduced total mitochondrial volume observed in the VMP1 KO cells (Fig. 5J). In this case, control and VMP1 KO cells had a similar total volume of mitochondria following LPS and ATP treatment (Fig. 5J). Previous work suggested that altered mitochondrial fusion can result in mitochondrial fragmentation and increased apoptotic cell death [36]. Our data support that there may be a defect in mitochondrial fusion in treated VMP1 KO cells given that there was an increase in the number of mitochondria, a reduction in average mitochondrial volume as well as increased cell death in VMP1 KO cells following LPS and ATP treatment (Fig. 5I–J, 1F).

### VMP1 KO cells have increased levels of exposed cytoplasmic mtDNA and extracellular mtDNA.

**Figure 6:**
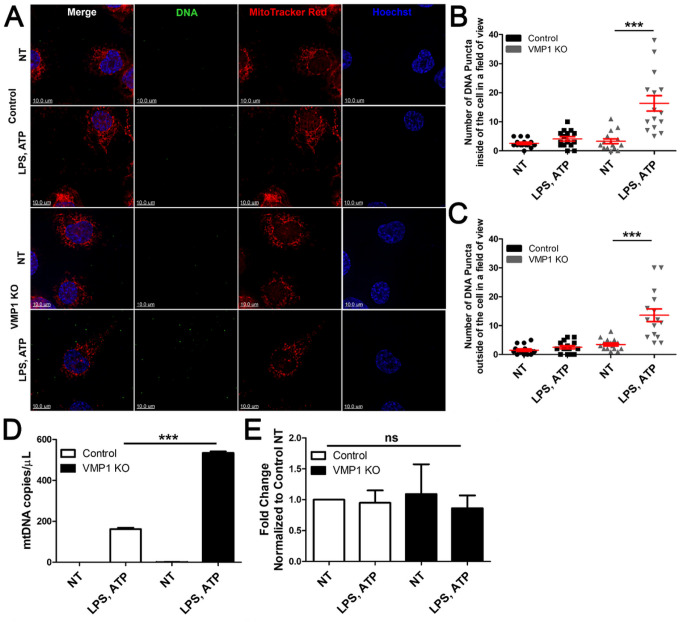
LPS and ATP treated VMP1 KO cells have more exposed cytoplasmic and released extracellular mtDNA than control cells. VMP1 KO or control THP-1s were treated with LPS and ATP then analyzed as follows: A) Immunofluorescence microscopy images of cells stained with MTRed dye and anti-DNA antibody. B) Quantification of the number of DNA+ puncta inside of the cell. C) Quantification of the number of DNA+ puncta outside of the cell. Data were quantified from 15 images per treatment group. D) qPCR data quantifying the number of mtDNA copies/μL in the supernatant. E) qPCR data representing the fold change of mtDNA/β2-microglobulin in the cell lysate. Data are representative of at least three independent experiments. Data are represented as mean ± SEM. Statistical differences were calculated using one-way ANOVA with Bonferroni post-test. For all statistical tests, *, **, ***, p < 0.05, 0.01, and 0.001, respectively.

When mitochondria are dysfunctional, mitochondrial outer membrane permeabilization (MOMP) occurs, allowing for the extrusion of the mitochondrial inner membrane into the cytosol and release of mtDNA [37]. To determine whether there was increased exposure of cytoplasmic mtDNA in VMP1 KO cells, differentiated THP-1s were treated then incubated with MTRed and stained with anti-DNA antibodies under mild detergent conditions to prevent the staining of mtDNA in intact mitochondria but to detect cytoplasmically exposed mtDNA (Fig. 6A, S4A,B). Representative images showed that treatment of VMP1 KO cells with LPS and ATP led to an increase in cytoplasmic DNA puncta, but there also appeared to be an increase in DNA pucta attached to the glass coverslip outside of the boundary of the cell (Fig. 6A). Imaris software was utilized to quantify the number of DNA puncta inside and outside of the cell (Fig. S5). The data demonstrated that there were relatively few DNA puncta inside of the cell in untreated control and VMP1 KO cells and control cells treated with LPS and ATP This contrasts with the larger increase in the number of intracellular DNA puncta per field of view for LPS and ATP treated VMP1 KO cells (Fig. 6B). Interestingly, it was observed that there was also an increase in DNA puncta on the coverslips outside of the cell in VMP1 KO cells treated with LPS and ATP (Fig. 6C). To validate this result, we isolated DNA from the culture supernatant and quantified cell-free mtDNA via qPCR using primers specific to genes expressed in the small, circular mitochondrial genome. Using this assay, we detected mtDNA in the supernatant of LPS and ATP treated control cells, and a further increase in mtDNA in the supernatant from LPS and ATP treated VMP1 KO cells (Fig. 6D). There was no difference in the amount of mitochondrial DNA across conditions in the cell lysates even though there were more mitochondria in VMP1 KO cells (Fig. 6E, 5I). Overall, these data suggested that LPS and ATP treatment in VMP1 KO cells caused the exposure of cytoplasmic mtDNA and the release of extracellular mtDNA which is likely to contribute to the increased inflammatory responses observed in these cells.

### VMP1 KO cells have disrupted autophagic flux.

**Figure 7:**
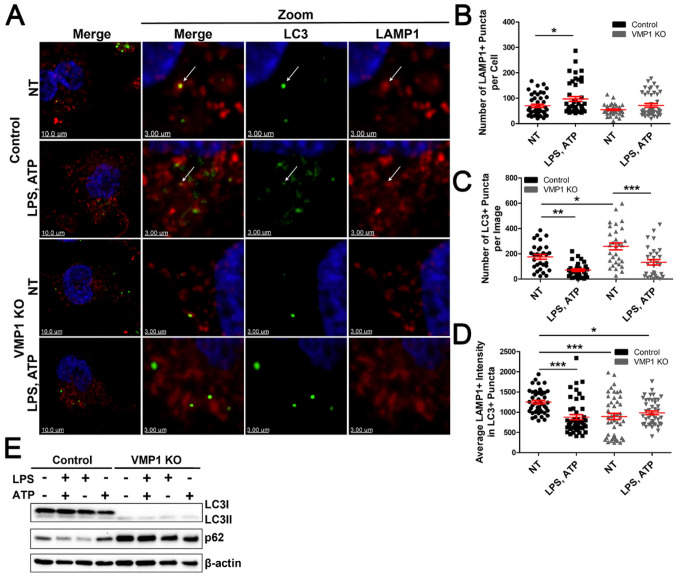
VMP1 KO cells have disrupted autophagic flux Differentiated control or VMP1 KO cells were untreated or treated with LPS and ATP A) Representative images from control or VMP1 KO cells untreated or treated with LPS and ATP then stained for LAMP1 and LC3. The white arrows point to colocalization of LAMP1 and LC3. B) Quantification of the number of LAMP1+ puncta per cell. C) Quantification of the number of LC3+ puncta per image. D) Quantification of the average LAMP1 intensity per image in LC3+ puncta. Data are from at least three independent experiments with at least 10 images per condition. E) LC3B and SQSTM1/p62 levels were probed by western blot. Statistical differences were calculated using one-way ANOVA with Bonferroni post-test or Dunnett’s multiple comparison test. For all statistical tests, *, **, ***, p < 0.05, 0.01, and 0.001, respectively.

Given that autophagy modulates inflammatory responses and that previous reports have suggested that VMP1 KO can impair autophagic flux, we wanted to assess autophagic processes in VMP1 KO macrophages under basal and inflammatory conditions [11, 38, 39]. Double staining or colocalization of microtubule-associated protein 1 A/1 B-light chain 3 (LC3) and lysosome-associated membrane protein 1 (LAMP1) suggested fusion of the autophagosome and lysosome which was primarily observed in control cells (Fig. 7A). The number of LAMP1 + puncta were about the same between control and VMP1 KO with a slight increase in control cells treated with LPS and ATP (Fig. 7B). There was a modest but significant increase in the number of LC3 + puncta in untreated VMP1 KO cells compared to untreated control cells (Fig. 7C). Interestingly, the number of LC3 + puncta with LPS and ATP treatment for both control and VMP1 KO cells decreased (Fig. 7C). Untreated control cells exhibited the highest level of colocalization between LC3 and LAMP1, suggesting fusion of the autophagosome and lysosome which was disrupted in treated control cells and untreated and treated VMP1 KO cells (Fig. 7D) which was consistent with previous reports [5, 38]. Notably, while image analysis revealed what appeared to be modest changes in autophagic function in VMP1 KO cells, immunoblotting results demonstrated a marked increase in LC3II levels in VMP1 KO cells and an accumulation of p62 compared to control cells suggesting that autophagic flux was disrupted (Fig. 7E). Notably, there was an absence of detectable LC3I in VMP1 KO cells which is supported by previous reports that LC3II levels can increase even under conditions when autophagy is blocked (Fig. 7E) [40]. Taken together, these data suggested that autophagy was disrupted in VMP1 KO which is consistent with an accumulation of damaged mitochondria and extracellular mtDNA which is was hypothesized to be released through secretory autophagy.

## Discussion

In this study, we used genetic deletion of VMP1, a protein previously identified for its critical role in regulating SERCA activity [41] and autophagic flux [5], to elucidate whether VMP1 regulates innate inflammatory responses. This study is the first to characterize the role of VMP1 in innate immune responses and autophagic processes. Initial experiments demonstrated that VMP1 negatively regulates the release of IL-1β gal-3 in response to canonical NLRP3 agonists, as VMP1 KO led to increased release of IL-1β and gal-3 (Fig. 1 and S1C). Increased release of IL-1β in response to LPS and ATP suggested this phenotype was due in part to increased inflammasome activation. VMP1 KO macrophages had exacerbated NLRP3 inflammasome activation in response to LPS and ATP (Fig. 1). The NLRP3 inflammasome is uniquely activated by a variety of seemingly unrelated signals, and our data showed that several signals were increased in VMP1 KO cells [42]. These signals included Ca^2+^ mobilization, mitochondrial dysfunction, and release of mtDNA (Fig. 3, 5, and 6). Ca^2+^ mobilization as a signal upstream of NLRP3 inflammasome activation is likely to be indirect, either leading to increased potassium efflux [43] or alterations of mitochondrial function. Our data demonstrated that in control THP-1s treated with thapsigargin, an inhibitor of the Ca^2+^ ATPase SERCA, as well as LPS and ATP there was an increase in caspase-1 activation like what was observed in VMP1 KO cells that have diminished SERCA activity (Fig. 3E) [5, 44–46]. Our data suggested that Ca^2+^ mobilization alone was not sufficient to induce inflammasome activation, but impaired responses to changes in cytoplasmic [Ca^2+^] exacerbated responses to additional inflammatory stimuli. This dysregulated response to Ca^2+^ and Ca^2+^ overload likely contributed to mitochondrial dysfunction indicated by the loss of membrane potential and release of mtDNA (Figs. 5 and 6). Previous work has shown that NLRP3 activators can trigger apoptosis leading to the loss of mitochondrial membrane potential and release of mtDNA into the cytosol which can then trigger NLRP3 inflammasome activation [47]. We believe that the combination of these signals resulted in increased NLRP3 inflammasome activation in VMP1 KO cells (Fig. 1D,E).

Previous studies have demonstrated that in VMP1 KO cells autophagy was impaired at autophagosome/lysosome fusion [5, 38]. Our data was consistent with these findings where we found that in VMP1 KO cells there was less colocalization between LC3 and LAMP1 in untreated cells as well as decreased levels of LC3-I and increased p62 in treated and untreated cells (Fig. 7). This defect in autophagy likely resulted in the persistence of dysfunctional mitochondria and increased NLRP3 inflammasome activation (Fig. 5, 1 D,E) [11, 47–49]. Along these lines, previous work showed that caspase-1 activation resulted in the inhibition of mitophagy resulting in the release of mtDNA into the cytoplasm and more dysfunctional mitochondria [50]. Perhaps both VMP1 and caspase-1 activation regulate mitophagy such that with inflammatory stimulation and Ca^2+^ overload almost all mitochondria in VMP1 KO cells lose membrane potential and release more mtDNA into the cytosol (Fig. 4, 5, and 6). Typically, with NLRP3 activation, p62 is recruited to damaged mitochondria which are then ubiquitinated through a Parkin-mediated mechanism for degradation, but mitophagy is defective in the absence of VMP1 expression which causes an accumulation of damaged mitochondria [51, 52]. Additionally, another mechanism that restricts inflammasome activation involves the interaction between p62 and ASC which targets inflammasome components to autophagosomes for degradation although this response is likely defective in VMP1 KO cells [39]. Aside from promoting inflammasome activation, autophagy inhibition can elevate IL-1β release due to an increase in available proIL-1β for cleavage in the cytosol similar to what we observed (Fig. 1) [53]. As a result of impaired autophagic flux in VMP1 KO cells, we hypothesized that damaged mitochondria and mtDNA were released through secretory autophagy recently termed autophagic secretion of mitochondria [54]. Recent work suggested that mtDNA was primarily released from the cell due to membrane rupture, but a fraction of the mtDNA can also be released through GSDMD/gasdermin E (GSDME) pores upon pyroptotic/apoptotic cell death [55]. However, it seems more likely that in VMP1 KO cells due to diminished autophagosome fusion with the lysosome, there was increased fusion of the autophagosome containing damaged mitochondria and mtDNA with the plasma membrane to release these contents from the cell that otherwise could not be degraded (Fig. 6C,D) [56]. Cathepsin B which is released from damaged lysosomes had more activity in VMP1 KO cells and could increase NLRP3 inflammasome activation (Fig. 7F) [20, 57]. Overall, our data suggest that VMP1 through its interactions with SERCA and its modulation of autophagy dampen inflammatory responses by keeping a number of proinflammatory signals in check including Ca^2+^ mobilization, degradation of dysfunctional mitochondria, and the release of cytoplasmic and extracellular mtDNA.

Recently, a number of studies have found that VMP1 may be a host-factor utilized by viruses during infection [58–60], suggesting that VMP1 is a putative antiviral target. While our study does not preclude the possibility that VMP1 might be leveraged in this way, our findings do suggest that targeting VMP1 interactions with viral proteins may be more desirable than inhibiting VMP1 activity more broadly, which may have negative impacts on cellular inflammatory responses that may occur following VMP1 inhibition. This is supported by recent reports that have observed decreased expression of VMP1 has been identified in the monocytes of patients with Primary-Progressive MS and in the peripheral blood mononuclear cells (PBMCs) of PD patients [61, 62]. The progression of these neurologic diseases is characterized by dysregulated inflammatory responses that contribute to disease sequalae.

In summary, we showed that in response to inflammatory stimuli VMP1 KO cells release more IL-1β and gal-3 due at least in part to increased inflammasome activation compared to control cells. Following ATP stimulation, VMP1 KO cells had elevated levels of cytoplasmic [Ca^2+^] which resulted in the loss of membrane potential in almost all mitochondria and the cytoplasmic release of mtDNA. Impaired autophagic flux in VMP1 KO cells prevented the cells from degrading damaged mitochondria and promoted the release of mtDNA further exacerbating inflammatory responses. Taken together, our findings identify a novel role for VMP1 in modulating inflammatory responses. Our findings support a potentially critical role for VMP1 in the progression of these diseases that perhaps can one day be targeted therapeutically.

## Material And Methods

### Materials

#### Cell Culture, Differentiation, and Treatments.

HEK293T and THP-1 cells were obtained from the American Type Culture Collection (ATCC). Cells were cultured with 5% CO_2_ at 37°C in either DMEM or RPMI supplemented with 10% heat-inactivated fetal bovine serum (FBS) (Gibco),10 μg/mL ciprofloxacin hydrochloride, 100 IU/mL penicillin, and 100 μg/mL streptomycin. THP-1s were differentiated by adding phorbol 12-myristate 13-acetate (PMA) (Sigma-Aldrich, P1585) at a concentration of 1 μg/mL for 48 h. The cells were then allowed to rest for 72 h prior to treatment. Unless otherwise noted, the cells were treated with 100 ng/mL lipopolysaccharides from *Escherichia coli* O55:B5 (Sigma-Aldrich, L4524) for 4 h. The media was changed and then some wells were treated with 5 mM adenosine 5-triphosphate disodium salt hydrate (ATP) (Sigma-Aldrich, A2383) for 30 min. To measure the response to α-syn, α-syn fibrils were obtained as described previously, and 1 μM α-syn was added to differentiated THP-1s for 24 hrs [63]. To test SERCA inhibition, 1 μm thapsigargin (Tocris, 1138) was added at the same time as any inflammatory stimuli including when it served as a control. The supernatant or cells were then collected for analysis.

#### Cloning and generation of stable cell lines.

VMP1 knockout THP-1 cell lines were generated using a modified version of the LentiCRISPRv2 plasmid (Addgene plasmid number 52961, a gift from Feng Zhang) that has the puromycin resistance cassette replaced with a G418 resistance cassette to create LentiCRISPRv2-G418 [64]. The following oligonucleotide guide RNA sequence was annealed and cloned into LentiCRISPRv2-G418: VMP1 guide RNA 5’-CTTTTGTATGCCTACTGGAT-3’ as described previously [4, 23]. Cells transduced with the LentiCRISPRv2-G418 backbone served as a control for selection. To generate stable cell lines, lentivirus was prepared by transfecting equal amounts of VSV-G, psPAX2 (from Didier Trono, NIH AIDS Reagent program [catalog number 11348]) [65, 66], and LentiCRISPRv2-G418 (either the backbone or the clone containing the guide RNA of interest) using polyethylenimine (PEI) into HEK293T cells. Retrovirus was prepared by transfecting equal amounts of VSV-G, pCigB, and pMSCVpuro-Mito-Pericam using PEI into HEK293T cells. Viral supernatant was harvested 48 h posttransfection and filtered through 0.45-μm filters (Millipore). The concentrated supernatant was applied to THP-1 cells by spinoculation at 13°C for 2 h at 1,200 × g. Media was changed 24 h later. Forty-eight hours after transduction, geneticin (G418) (Gibco) was added to the cells at a concentration of 0.5 mg/mL. Following 3–4 weeks of selection, lymphocyte separation media was used to remove dead cells, and healthy cells were collected to validate the knockout by western blot. VMP1 KO cells were maintained in culture for at most 4 weeks following successful selection.

To measure caspase-1 activation, THP-1s were transduced with lentiviral vector prepared as described above with a caspase-1 biosensor containing the IQAD amino acid target sequence as described previously [28].

#### Fibrillar α-syn preparation.

Human wild-type α-syn was expressed in E. coli BL21 DE3 CodonPlus cells and purified as previously described [67]. Monomeric, endotoxin free (Pierce LAL Chromogenic Endotoxin Quantification Ki), α-syn (200μM) in 50mMTris-HCl, pH 7.5, 150mMKCl was assembled into fibrils by incubation at 37°C under continuous shaking in an Eppendorf Thermomixer set at 600 r.p.m. for 7 days [68]. The assembly reaction was monitored by thioflavin T binding, the nature of the fibrillar assemblies was assessed by transmission electron microscopy after negative staining with 1 % uranyl acetate and their proteolytic fingerprint verified by digestion by Proteinase K [68]. The resulting fibrils were centrifuged twice at 15,000 g for 10 min and re-suspended twice in PBS. Their concentration was adjusted to 350μM in PBS. They were next fragmented to an average length of 40–50 nm by sonication for 20 min in 2 mL Eppendorf tubes using a Vial Tweeter powered by an ultrasonic processor UIS250 v (250 W, 2.4 kHz; Hielscher Ultrasonic [69]. Fragmented fibrils were aliquoted (6 μL) in 0.5 mL Eppendorf tubes, flash frozen in liquid nitrogen and stored at − 80°C until use.

#### Sandwich ELISAs.

Cell culture supernatants were analyzed using the following kits: Human IL-1beta/IL-1F2 DuoSet ELISA (R&D Systems, DY201 −05) for IL-1β and Human Galectin-3 DuoSet ELISA for gal-3 (R&D Systems, DY1154). The manufacturers’ protocols were followed. Alternatively, gal-3 protein levels were also measured using an in-house sandwich ELISA described previously [70]. Briefly, mouse anti-LGALS3 B2C10 (Santa Cruz Biotechnology, SC-32790) was diluted in pH 9.6 carbonate buffer to a final concentration of 1 μg/mL to coat 96-well Maxisorp ELISA plate (Nunc, 44-2402-22) at 4°C overnight on an orbital shaker. Between each step the wells were washed 3x-5x with PBS containing Tween-20. The wells were blocked 1:1 with RPMI supplemented with 10% characterized FBS and PBS for 2 h at RT. The culture supernatant was then added to the wells. The standard curve was generated by serially diluting recombinant gal-3 (Abcam, ab89487). Biotin conjugated rat anti-LGALS3 (M3/38; Millipore Sigma, 125402) was diluted to a final concentration of 500 ng/mL in PBS with 1% bovine serum albumin (BSA) (Sigma-Aldrich, A7906) and added to the wells for 2 h on a rocker at RT. Then streptavidin HRP (ImmunoReagents, Ba-103-HRPX) was diluted to 1 μg/mL and incubated for 30 min at RT on a rocker.

The HRP signal was detected with the addition of 1 × 3,3’,5,5-tetramethylbenzidine (Invitrogen, 00-4201-56) and then the reaction was quenched with 2 N sulfuric acid. The absorbance was read at 450 nm on a PowerWave XS plate reader (BioTek Instruments) with Gen5 software. The standard curve was fit with a 4-Parameter Logistic (4PL) curve.

#### Quantitative real-time PCR.

Cells were treated as described above and RT-PCR was used to measure changes in gene expression. Total RNA was purified from cell lysates following treatment using the NucleoSpin RNA Plus extraction kit (Macherey-Nagel, 740984.250). cDNA was synthesized using the GoScript Reverse Transcriptase System (Promega, A5004). Quantitative PCR was performed using gene specific primers and Itaq^™^ Universal SYBR (Bio-Rad, 1725124). The following primer sets were used in this study: VMP1 fwd, 5’-GTGGCTTTCATTGGTGCTGTCC-3’; VMP1 rev, 5’-GAGTTCAACCGCTGCTGGATTC-3’; proIL-1β fwd, 5’ AATCTGTACCTGTCCTGCGTGTT-3’; proIL-1β rev, 5’-TGGGTAATTTTTGGGATCTACACTCT-3’; lgals3 fwd, 5’-GCCAACGAGCGGAAAATGG-3’; lgals3 rev, 5’-TCCTTGAGGGTTTGGGTTTCC-3’; GAPDH fwd, 5’-GCACCGTCAAGGCTGAGAAC-3’; GAPDH rev, 5’-G CCTTCTCCATGGTGGTGAA-3’.

GAPDH was utilized as a housekeeping gene for normalization.

#### RNA-Sequencing and pathway analysis.

Control or VMP1 KO differentiated THP-1s were left untreated or treated with 100 ng/mL LPS (4 h) then 5 mM ATP (30 min). Supernatant was collected and analyzed by gal-3 ELISA as described above to ensure that the sample phenotype was consistent with previous experiments. RNA was isolated using the NucleoSpin RNA Plus extraction kit. Part of the RNA was saved for qPCR to assess proIL-1β, and gal-3 gene expression as described above. RNA samples were submitted to the University of Chicago Genomics Facility to assess the concentration and quality of the RNA. Samples that passed the check were then used for library preparation and whole genome sequencing using Illumina NovaSeq. Pathway analysis was performed following a previously published protocol [71]. Heatmaps were generated using the pheatmap function (RRID:SCR_016418) in R.

#### Western blotting.

Protein was isolated by lysing pelleted cells using lysis buffer containing 100 mM Tris, pH 8.0, 1 % NP-40 (Thermo Fischer Scientific, 85124), 150 mM sodium chloride and Pierce protease inhibitor cocktail (Thermo Fischer Scientific, 32953) on ice for 30 min. The lysates were centrifuged at 4°C for 10 min at 10,000 × *g* and then the supernatant was collected and transferred to a new tube. The protein concentrations were determined by Pierce 660 nm protein assay (Thermo Fischer Scientific, 22660). In brief, 2x SDS was added to the lysed sample and boiled at 95°C for 5 min. An equal amount of protein was loaded into a 12% polyacrylamide gel or a precast 4–20% gradient polyacrylamide gel (Mini-PROTEAN TGX Precast Polyacrylamide Gels, Bio-Rad, 4561096) for SDS-PAGE. After separation, the proteins were transferred to a nitrocellulose membrane (Bio-Rad, 162 – 0115) and probed overnight at 4°C unless otherwise indicated with the primary antibody diluted in powdered milk block solution at 2.5 g/50 mL of Tris-buffered saline, 0.1 % Tween 20 (Sigma-Aldrich, P7949–500ML). The primary antibodies were rabbit anti-VMP1 (1:1000; StressMarq Biosciences, SPC-680D), mouse anti-β-actin (1:1000 at RT for 1 hr; Proteintech, 66009–1-Ig), rabbit anti-LC3B (1:1000; Sigma-Aldrich, L7543), and rabbit anti-p62 (1:500; Cell Signaling, 7695S).

The nitrocellulose membrane was washed in Tri–buffered saline, 0.1% Tween 20 and probed with horseradish peroxidase (HRP)-conjugated goat anti-mouse (Thermo Fischer Scientific, 12–349) or HRP-conjugated anti-rabbit secondary antibody (Thermo Fisher Scientific, 12–348) diluted in milk block solution at 1:10000 for 30 min. HRP was detected with the addition of SuperSignal West Fempto Chemiluminescent Substrate (Thermo Fischer Scientific, PI34096). Chemiluminescence levels were measured using the FluorchemE Imaging System (Protein Simple).

#### Caspase-1 activation assays.

For the FAM-FLICA caspase-1 activation assay, following treatment, the cells were incubated with FAM-FLICA caspase-1 (YVAD) substrate following the manufacturer’s protocol (Immunochemistry Technologies, 97). Briefly, the FLICA substrate that was resuspended in DMSO was diluted in PBS 1:5. The diluted substrate was added to the wells at a final concentration of 1:30. The plate was incubated at 37°C for 1 h. The cells were washed with apoptosis buffer from the kit and were allowed to sit for 10 min in the incubator. Hoechst dye was added at a dilution of 0.5% in apoptosis buffer. The cells were incubated with the dye for 15 min then the cells were washed once with 1x apoptosis buffer then fresh buffer was added prior to imaging. A 20x lens was used to take 10 images per well. Data were collected by z-stack imaging and were analyzed as maximum intensity projections (MIPs). Cells were imaged using z-stacks with 1 μm between each stack and a total of 5 z-stacks. A surface algorithm was built in Imaris around each individual cell, and the data are displayed as the intensity max of each individual cell for a given treatment.

To measure caspase-1 activation using the biosensor, following treatment, cells were lysed with 1x passive lysis buffer (Promega, E1941). Lysates were transferred to a white 96 well plate in duplicate or triplicate. Firefly luciferase substrate was added, and luminescence (relative light units) was quantified.

#### Lactate dehydrogenase assay.

Lactate dehydrogenase (LDH) release was measured using a previously published protocol [70, 72]. To measure LDH release, supernatant was collected 3 h after signal 2. The samples were plated in duplicate in a clear 96-well plate. Signal was measured by reading the absorbance at λ = 490 nm on a Synergy HTX Multi-Mode plate reader (BioTek Instruments) with Gen5 software.

#### Calcium live cell imaging.

To measure [Ca^2+^]_cyt_, cells were incubated at RT with the high affinity Ca^2+^ indicator Fluo-4/AM (ThermoFisher Scientific, F14201) for 15 min in Tyrode’s solution (NaCl 135 mM; KCl 4 mM; CaCl_2_ 3 mM; MgCl_2_ 1 mM; glucose 10 mM; HEPES 10 mM; pH 7.4). Fluo-4 was measured at an excitation/emission of 488/>515 nm. Cells were perfused with Tyrode’s solution for 2 min for a baseline recording. Then cells were perfused with 5 mM ATP in Tyrode’s solution for 1 min or 5 μM thapsigargin (Tocris, 1138) in Tyrode’s solution without CaCl_2_ for 2 min. Once the recording returned to baseline, 2 μM ionomycin (Sigma-Aldrich) in Tyrode’s solution was added to reach F_max_. A laser scanning confocal microscope (Radiance 2000 MP Bio-Rad, UK) equipped with a 40x oil-immersion objective lens (N.A.=1.3) was used to record changes in cytosolic [Ca^2+^]_cyt_. Fluo-4 recordings were acquired in line-scan mode (3 ms per scan; pixel size 0.12 μm). All images were analyzed using ImageJ software (NIH, USA). The [Ca^2+^]_cyt_ was calculated by the following formula: [Ca^2+^]_cyt_ = (F_0_ – F_min_)/(F_max_ – F_min_), where F_0_ was the Fluo-4 fluorescence; F_max_ and F_min_ were the fluorescence level at 3 mM Ca^2+^/ionomycin and at the lowest baseline recording, respectively. The calcium-induced calcium release was calculated as the summation of the area under the curve for 150 seconds for ATP and 900 seconds for thapsigargin reported in arbitrary units.

To measure [Ca^2+^]_mito_, either control or VMP1 KO THP-1s were transduced with retroviral vector containing a pMSCVpuro-Mito-Pericam construct as described above [33]. pMSCVpuro-Mito-Pericam was a gift from Bjorn Stork (Addgene plasmid #87381, http://n2t.net/addgene:87381; RRID:Addgene_87381). THP-1s were differentiated in delta T dishes (Bioptechs, 04200417B). Prior to imaging, the media was replaced by Tyrode’s solution (NaCl 135 mM; KCl 4 mM; CaCl_2_ 3 mM; MgCl_2_ 1 mM; glucose 10 mM; HEPES 10 mM; pH 7.4). Images were acquired using the 60x lens with the EMCCD camera. Cells were excited at 380 nm (DAPI excitation) or 495 nm (FITC excitation) and emission was recorded at 510 nm [73]. Baseline recordings were taken for approximately 30 sec prior to the addition of 5 mM ATP Five points were taken per dish, and recordings were taken every 8 sec for about 5 min. All images were analyzed using ImageJ software (NIH, USA). The influx rate was quantified for each cell by using the slope of the linear fit of the fluorescence change during 15 sec following the addition of ATP The efflux rate was quantified for each cell using the slope of the linear fit of a 50 sec period after calcium levels started to decline.

#### MitoTracker live cell imaging.

To assess mitochondrial mass and mitochondrial membrane potential, THP-1s were differentiated in delta T dishes (Bioptechs, 04200417B) and treated as described above. Following treatment, cells were incubated with a final concentration of 100 nM MitoTracker Red CMXRos (Invitrogen, M7512), 50 nM MitoTracker Green FM (Cell Signaling Technology, 9074S), and 67 ng/mL Hoechst 33342 (ImmunoChemistry Technologies, 639) in serum free RPMI media for 20 min in the incubator. The cells were washed once with FluoroBrite^™^ DMEM (Gibco, A1896701) supplemented with 10% FBS and 4 mM L-glutamine (Thermo Fisher Scientific, 25030081). Prior to imaging, the heat chamber was set to 37°C and supplemented with 5% CO_2_. The exposure conditions were determined based on the treatment conditions where it was expected for there to be the lowest intensity of MitoTracker Red. For each dish, 20 images were acquired with 20 z-stacks per image at 0.5 μm per z-stack for 10 μm total.

#### Immunofluorescence imaging.

THP-1s were differentiated onto glass coverslips and treated as described above. To visualize mtDNA in the cytoplasm, following treatment, cells were incubated with a final concentration of 100 nM MitoTracker Red CMXRos (Invitrogen, M7512) in serum free RPMI media for 30 min in the incubator. Cells were then washed once with serum free RPMI media then were fixed with 3.7% formaldehyde, methanol free (Polysciences, 04018–1) in 0.1 M piperazine-N-Nbis[2-ethanesulfonic acid] (PIPES) buffer for 10 min. Cells were then washed 3x with PBS. The cells were permeabilized with 0.1 % saponin in PBS block solution supplemented with 10% NDS and 0.01 % sodium azide and incubated with mouse anti-DNA clone AC-30–10 (1:25, Millipore Sigma; CBL186) for 1 h at RT. Cells were then washed 3x with PBS. Cells were then incubated with 100 ng/mL Hoechst 33342, 488 conjugated donkey anti-mouse (1:300; Jackson ImmunoResearch Laboratories, 715-545-150), and phalloidin-iFluor 647 reagent (1:1000, Abcam; ab176759) for 20 min at RT. Cells were then washed 3x with PBS and were mounted onto coverslips. For each coverslip, 15 images were acquired with 20 z-stacks per image at 0.5 μm per z-stack for 10 μm total.

To visualize LC3/LAMP1, cells were fixed with 3.7% formaldehyde, methanol free in 0.1 M PIPES buffer for 10 min. Cells were then washed 3x with PBS. The cells were permeabilized with 0.1 % saponin in PBS block solution supplemented with 10% NDS and 0.01 % sodium azide and incubated with rabbit anti-LAMP1 (1:1000, Abcam; ab24170) and mouse anti-LC3B (E5Q2K) (1:300, Cell Signaling; 83506) for 1 h at RT. Cells were then washed 3x with PBS. Cells were then incubated with 100 ng/mL Hoechst 33342, 488 conjugated donkey anti-mouse (1:300), 647 conjugated donkey anti-rabbit (1:300. Jackson ImmunoResearch Laboratories; 711-605-152) for 20 min at RT. Cells were then washed 3x with PBS and were mounted on coverslips. For each coverslip, 15 images were acquired with 20 z-stacks per image at 0.5 μm per z-stack for 10 μm total.

#### Wide-field fluorescence deconvolution microscopy.

Cells were imaged on a DeltaVision wide-field fluorescence microscope (Applied Precision, Inc.). It has a digital camera (CoolSNAP HQ2; Photometrics). An oil immersion Olympus Plan Apo 60x objective lens (N.A.=1.42), Olympus UplanSApo 100x objective lens (N.A. = 1.40), or Olympus LUCPlanFLN 20x objective lens (N.A. = 0.45) was coated with 1.518 refraction index low autofluorescence immersion oil, Olympus Type F (Fischer Scientific, NC0297589). A 250 watt Xenon Arc lamp was used to direct excitation lighting from the back of the microscope and focused from below onto the coverslip held on an Olympus IX-71 stage. Dichroic filter set uses the Alexa setting: FITC excitation: 475/28 Emission: 523/36; A594 excitation: 575/25 emission: 632/30; CY5 excitation: 632/20 emission: 67634; DAPI excitation: 390/18 emission: 435/38. Exposure times varied depending on the type of experiment and staining conditions.

#### Image analysis.

The collected z-stack images were used as reconstructed 3-dimensional MIPs for analysis with Imaris software (version 7.6.4, Bitplane) specifically the 3-dimensional masking algorithm function. The same masking algorithm was applied to all images and conditions of a single experiment to allow for consistent group comparisons using the Batch Coordinator tool (Imaris, Bitplane). For the MitoTracker live cell assay, the spots algorithm was built around the MTGreen signal with an estimated diameter of 0.750 μm. For the mtDNA immunofluorescence imaging, a surfaces algorithm was built around the 647 phalloidin channel with the diameter equal to 20 μm and volume above 78.5 μm^3^ to create two new channels one for inside of the cell and one for outside of the cell. A spots algorithm was then built around the 488 DNA signal with an estimated diameter of 0.400 μm and volume between 0.500 μm^3^ and 2.000 μm^3^. For the LC3/LAMP1 immunofluorescence imaging, a spots algorithm was built around the 488 LAMP1 signal with an estimated diameter of 0.400 μm and area above 0.100 μm^2^. Another spots algorithm was built around the 647 LC3 signal with an estimated diameter of 0.500 μm and area above 0.500 μm^2^.

#### Quantification of cellular and cell-free mtDNA using qPCR.

Quantitative PCR was performed to measure both cellular and cell-free mtDNA as described in a published protocol [74]. Briefly, cells were treated as described above then the supernatant and cell pellets were collected. The supernatant was spun down at 1500 rpm for 10 min at 4°C to remove cell debris. The cell pellets were resuspended in 200 μL PBS. DNA was isolated following the manufacturer’s protocol from the QIAamp DNA Mini Kit (Qiagen, 51304) except the samples were lysed with buffer AL, mixed by pulse-vortexing, and were incubated at 56°C for 10 min. The DNA was then placed in a bath sonicator for 5 min for supernatant or 10 min for cell lysate. After sonication, the concentration of DNA in each sample was determined and was adjusted to the same concentration. The standard curves were generated as described. The quantity of mtDNA in the supernatant is reported as the absolute copy number per μL. The quantity of mtDNA in the cell lysate is reported as the fold difference using the formula 2^(-ΔΔC^t^)^.

### Statistical Analysis.

Data were analyzed using GraphPad Prism 5.0. Unless otherwise stated, graphs are presented as the mean ± SEM of at least three independent experiments. Statistical differences were calculated using one-way or two-way ANOVA with Bonferroni post-test, repeated measures ANOVA with Bonferroni post-test, or two-tailed unpaired t-test as indicated in the figure legends. P-values < 0.05 were considered significant and are designated by: * *p*< 0.05, ** *p*< 0.01, *** *p*< 0.001.

## Abbreviations

4PL: 4-Parameter logistic
α-syn: α-synuclein
ATP: Adenosine 5-triphosphate disodium salt hydrate
AD: Alzheimer’s Disease
CARD: ASC Apoptosis-associated speck-like protein containing a
ALP: Autophagic/lysosomal pathway
BSA: Bovine serum albumin
DAMP: Damage-associated molecular pattern
ER: Endoplasmic reticulum
FBS: Fetal bovine serum
FLICA: Fluorochrome-labeled inhibitors of caspases
Gal-3: Galectin-3
GSDMD: Gasdermin D
GSDME: Gasdermin E
G418: Geneticin
HRP: Horseradish peroxidase
KO: Knock out
LDH: Lactate dehydrogenase
LPS: Lipopolysaccharide
LAMP1: Lysosome-associated membrane protein 1
LC3: Microtubule-associated protein 1A/1B-light chain 3
MIPs: Maximum intensity projections
mtDNA: Mitochondrial DNA
MOMP: Mitochondrial outer membrane permeabilization
MTGreen: MitoTracker Green FM
MTRed: MitoTracker Red CMXRos
MS: Multiple sclerosis
NLR: Nod-like receptor
NLRP3: Nucleotide-binding domain, leucine-rich repeats containing family, pyrin domain-containing-3
PD: Parkinson’s Disease
PAMPs: Pathogen associated molecular patterns
PBMCs: Peripheral blood mononuclear cells
PMA: Phorbol 12-myristate 13-acetate
PIPES: Piperazine-*N*-*N*bis[2-ethanesulfonic acid]
PEI: Polyethylenimine
ROS: Reactive oxygen species
SERCA: Sarcoplasmic/endoplasmic reticulum calcium ATPase
TLR: Toll-like receptor
VMP1: Vacuole membrane protein 1
